# Trends in HIV infection in the First Affiliated Hospital of Harbin, China

**DOI:** 10.1186/s12879-014-0605-1

**Published:** 2014-11-25

**Authors:** Hua-Feng Xu, Hai-Zhou Zhou, Li-Xin Jiang, Na Zhang, Xuan Zhang, Xiu-Ru Guan

**Affiliations:** School of Life Sciences, Heilongjiang University, Harbin, 150081 Heilongjiang China; Department of Laboratory Diagnosis, the First Affiliated Hospital of Harbin Medical University, 23 Youzheng Street, Nangang District, Harbin, 150001 Heilongjiang China

**Keywords:** HIV testing, Early diagnosis, Inpatients, Hospital, China

## Abstract

**Background:**

Major hospitals in most Chinese cities have the capability to perform HIV testing. However, it is not a routine test for all patients and, as a result, many patients are not aware of their HIV status. To understand the rate of HIV infection and the factors associated with infection, we tested serum to determine HIV status and analyzed factors associated with HIV infection.

**Methods:**

We collected blood samples from 348,151 patients who visited the First Affiliated Hospital of Harbin Medical University from 1 January 2007 to 31 December 2012. Serum was screened with an ELISA. If the test was positive, we conducted two additional ELISAs: a repeat with the initial kit and one with another kit. If there was a positive result with either of these two ELISA kits, western blotting was performed at Harbin Centers for Disease Control and Prevention.

**Results:**

The HIV positivity rate of inpatients significantly increased during the course of this study. HIV infection in patients appeared to differ by sex, age, occupation, marital status, educational level, and route of infection. HIV was more prevalent in men than in women. More than 80% of HIV-positive patients had not received higher (>12 years) education. From 2007 to 2012, HIV-positive patients were mainly infected through sexual transmission. For sexually acquired infections, the rate of HIV infections through homosexual contact has increased rapidly in recent years, and ranged from 36.4% to 65.1%.

**Conclusions:**

The number of patients diagnosed as HIV positive has increased in recent years. Offering routine HIV testing in hospitals is feasible and can increase linkage to HIV care and treatment for many individuals.

**Electronic supplementary material:**

The online version of this article (doi:10.1186/s12879-014-0605-1) contains supplementary material, which is available to authorized users.

## Background

By the end of 2011, it was estimated that 780,000 (620,000–940,000) people were living with HIV/AIDS in China, of whom 154,000 (146,000–162,000) were living with AIDS [1]. Despite the increased availability of drugs against HIV, the AIDS epidemic continues to grow in China [2]. To halt it, more needs to be done to prevent the spread of HIV. Education about safe sex can help, but increasing HIV testing is also of paramount importance [3]-[5]. HIV testing is the only way to ensure that all those who do not know they are infected have an opportunity for early diagnosis and treatment. Most people do not get tested because they know little about HIV. For example, it is estimated that 17% of Chinese people do not know that HIV exists [6]. Worldwide, >90% of HIV-infected individuals are estimated to be unaware of their status [7]. HIV today is a manageable chronic condition, and early diagnosis is essential for good prognosis [8]-[10]. Through HIV testing, uninfected individuals can take steps to avoid becoming infected, while infected individuals can avoid transmission to sexual partners or children. Currently, HIV tests are available in major Chinese hospitals but are not routinely offered. With so many people unaware of their HIV status, there is a clear need for routine HIV testing of hospitalized patients throughout China.

The First Affiliated Hospital of Harbin Medical University is the largest teaching hospital in Heilongjiang province. In 2009, the hospital had >1.8 million patient visits, and 58,000 patients underwent surgery. Routine HIV testing identified a large number of undiagnosed HIV infections and HIV-discordant partnerships among patients and their families.

## Methods

### Ethical statement

Written informed consent was obtained in the local language from all participants. The First Affiliated Hospital of Harbin Medical University Ethics Review Committee granted ethical approval for the study.

### Data collection and methods

Data from all patients that accepted routine HIV testing from 1 January 2007 to 31 December 2012 were included. HIV test requisition data included name, age, sex, patient registration number, blood collection date, ward, clinical department, diagnosis, and requesting physician. Additional data collected from HIV-positive patients included educational level, occupation, marital status, address, and telephone number. The data was captured with questionnaires. The docimaster of clinical laboratory filled some basic informations of patients. The interviewer of CDC filled sensitive data like the history of risk behavior and history of HIV in the family. All patients attended HIV screening on a voluntary basis. The interview schedule included sensitive data such as history of risk behavior and HIV in the family. It is possible that the data reported here were biased owing to recall error and the social stigma faced by HIV-positive patients. A few HIV-positive patients did not provide truthful information. In addition, 1% of patients who were positive after routine HIV testing did not undergo an HIV confirmation test. However, this small proportion of the sample may not have influenced the outcome of the study.

Peripheral blood samples were taken using 3.5-ml separation gel vacuum tubes. Serum preparation was done from all of the peripheral blood samples by repeated centrifugation for 15 min at 1,200 g. The serum was first tested with an ELISA. The testing consisted of screening with either the Anti-HIV 1 + 2 Antibodies ELISA Diagnostic Kit (LIVZON Diagnostics, Zhu Hai, China) or the Diagnostic Kit for Antibody to Human Immunodeficiency Virus (INTEC Products, Xia Men, China). If the screening test was negative for HIV antibodies, the test was complete. If the screening test was positive, additional testing was needed to make sure that it was not a false-positive result. For this additional test, we obtained a fresh blood sample and repeated the ELISA using both kits. If there was a positive result for either ELISA, then confirmatory western blotting was performed at Harbin Centers for Disease Control and Prevention. If the western blotting was positive, the person was considered to be infected with HIV. Patients needed to bring their personal ID card to obtain the western blotting results.

### Statistical analysis

We evaluated the relationship between the demographic characteristics of HIV-positive patients and the prevalence of HIV. All data were stored in a database (Microsoft Excel 2007) and all statistical analyses were performed using SPSS version 16.0 (Chicago, IL, USA). Categorical variables were analyzed using the *χ*^2^ test. Statistical significance was defined as *P* < 0.05.

## Results

**Figure 1:**
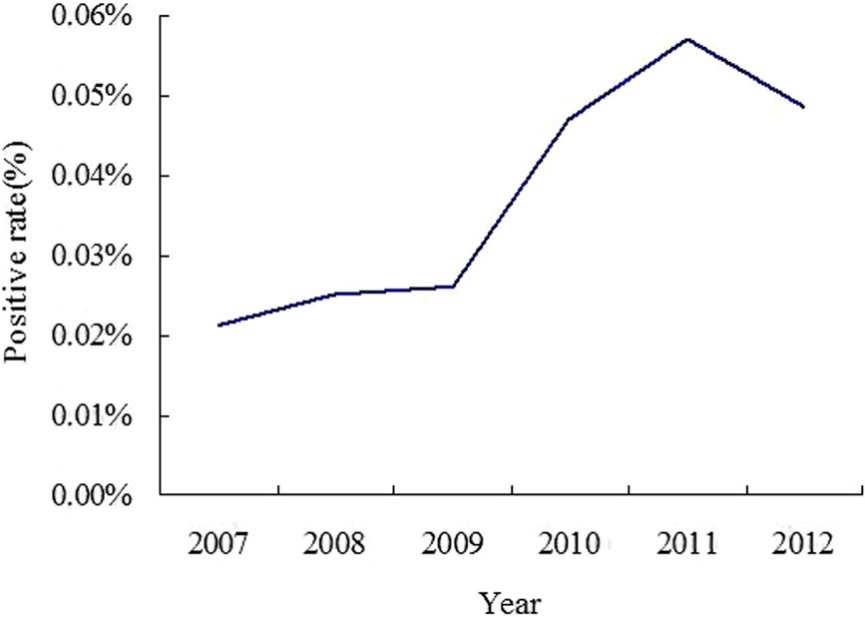
**The positive rate of HIV-1 testing from 2007 to 2012 in hospital.**

Yearly HIV testing volumes were 33,025, 43,891, 53,859, 59,493, 71,772 and 86,111. There was an increasing trend in the positivity rate of HIV-1 testing, from 0.2% (7/33,025) in 2007 to 0.5% (43/86,111) in 2012 (Figure 1).

### Demographic characteristics of HIV-positive patients

In 2007 and 2008, >50% of HIV-positive patients were 30–40 years old, whereas from 2009 to 2012 the age of HIV-positive patients showed a generally uniform distribution across the age groups from 20 to 60 years. More than 85% of the HIV-positive patients were 20–60 years old (*P* < 0.01) (Table 1). Additionally, in recent years some patients aged <20 years were HIV positive. Among HIV-positive patients, there was a significant difference according to sex, with men making up a significantly higher proportion of the HIV-positive patients than women (*P* < 0.05).

**Table 1:** **Demographic characteristics of HIV-infected inpatients**

	2007		2008		2009		2010		2011		2012		
	n = 7		n = 11		n = 14		n = 29		n = 42		n = 43		*P*
	n	%	n	%	n	%	n	%	n	%	n	%	
Age groups, years													
20	0	0.0%	0	0.0%	0	0.0%	1	3.5%	3	7.1%	3	7.0%	
20-60	5	85.7%	7	90.9%	8	92.9%	13	89.6%	36	85.8%	39	90.7%	
60	1	14.3%	1	9.1%	1	7.1%	2	6.9%	3	7.1%	1	2.3%	<0.01
Sex													
Male	4	57.1%	9	81.8%	10	71.4%	24	82.8%	35	83.3%	41	95.4%	
Famale	3	42.9%	2	18.2%	4	28.6%	5	17.2%	7	16.7%	2	4.7%	<0.05
Marry status													
No married	2	28.6%	2	18.2%	3	21.4%	9	31.0%	16	38.1%	14	32.6%	
Divorce or Widowed	3	42.9%	1	9.1%	6	42.9%	17	58.6%	15	35.7%	26	60.5%	
Married	2	28.6%	7	63.6%	3	21.4%	3	10.3%	10	23.8%	3	7.0%	
Unknown	0	0.0%	1	9.1%	2	14.3%	0	0.0%	1	2.4%	0	0.0%	<0.01
Occupation													
Students	0	0.0%	0	0.0%	0	0.0%	1	3.5%	1	2.4%	2	4.7%	
Unemployed	1	14.3%	1	9.1%	8	57.1%	2	6.9%	5	11.9%	6	14.0%	
Blue collar	6	85.7%	5	45.5%	1	7.1%	16	55.2%	26	61.9%	34	79.1%	
White collar	0	0.0%	0	0.0%	0	0.0%	1	3.5%	2	4.8%	1	2.3%	
Unknown	0	0.0%	5	45.5%	5	35.7%	9	31.0%	8	19.1%	0	0.0%	<0.01
Eduction, years													
6	0	0.0%	2	18.2%	2	14.3%	4	13.8%	6	14.3%	1	2.3%	
6-9	2	28.6%	5	45.5%	10	71.4%	10	34.5%	16	38.1%	14	32.6%	
10-12	4	57.1%	4	36.4%	2	14.3%	10	34.5%	13	31.0%	15	34.9%	
>12	1	14.3%	0	0.0%	0	0.0%	5	17.2%	7	16.7%	13	30.2%	<0.01
Routes of infection													
Blood/Drug	1	14.3%	1	9.1%	2	14.3%	7	24.1%	0	0.0%	3	7.0%	
Heterosexual	3	42.9%	6	54.6%	5	35.7%	6	20.7%	19	45.2%	12	27.9%	
Homosexual	3	42.9%	4	36.4%	7	50.0%	15	51.7%	21	50.0%	28	65.1%	
Unknown	0	0.0%	0	0.0%	0	0.0%	1	3.5%	2	4.8%	0	0.0%	0.12

With the exception of 2008, each year, more than two-thirds of HIV-positive patients described themselves as single (Table 1). HIV infection was associated with occupation, with a high percentage of HIV-positive patients being blue-collar workers (*P* < 0.01). Additionally, starting in 2010, HIV infection was found in white-collar workers and students. More than 80% of HIV-positive patients had not received higher education (Table 1), but the proportion of HIV-positive patients with higher education increased in the last 3 years of this study to 17.2%, 16.7% and 30.2% (*P* < 0.01).

No significant differences were found in the routes of infection. The yearly percentage of HIV-positive patients estimated to have been infected through sexual transmission ranged from 72.4% to 93.0%, and the proportion of HIV infections that were acquired through homosexual contact ranged from 36.4% to 65.1% (*P* > 0.05) (Table 1).

### Proportion of HIV-positive patients diagnosed in the different hospital departments

HIV-positive patients were divided into three groups based on the location in the hospital where they were tested: the sexually transmitted disease (STD) clinic, preoperative detection, and clinical detection. The STD clinic identified the smallest proportion of HIV-positive patients (Table 2). The number of patients diagnosed with HIV infection increased yearly because of preoperative and clinical detection. In the preoperative detection group, the number of HIV-positive patients diagnosed was three times higher in 2012 than in 2007. In the clinical detection group, the number of HIV-positive patients was 5.8 times in higher in 2012 than in 2007.

**Table 2:** **The source of HIV-infected inpatients**

	2007		2008		2009		2010		2011		2012	
	n = 7		n = 11		n = 14		n = 29		n = 42		n = 43	
	n	%	n	%	n	%	n	%	n	%	n	%
STD clinic	0	0.0%	1	9.1%	4	28.6%	4	13.8%	12	28.6%	8	18.6%
Preoperative detection	3	42.9%	6	54.6%	8	57.1%	8	27.6%	8	19.1%	12	27.9%
Clinical detection	4	57.1%	4	36.4%	2	14.3%	17	58.6%	22	52.4%	23	53.5%

## Discussion

The First Affiliated Hospital of Harbin Medical University is the largest teaching hospital in Heilongjiang province. In 2010, the hospital began to recommend routine HIV testing for all inpatients. We found that the HIV positivity rate of inpatients significantly increased (Figure 1), and that most of the patients did not know their HIV status. Unrecognized HIV infection is a persistent problem, resulting in continued transmission and morbidity of this devastating disease [11]. While HIV testing has long been a cornerstone of HIV prevention, and is currently available in most big hospitals in Chinese cities, it is not routinely offered. Knowing patients’ HIV status is important for patients, doctors and nurses. For those who test HIV positive, this knowledge may provide them with greater opportunities to access the services and support that will help manage their health and prolong life. Doctors and nurses are at risk of occupationally acquired infection from contact with the blood and other body fluids of HIV-positive inpatients [12],[13]. Awareness of HIV status would facilitate suitable precautions, such as protective eyewear and double gloving, to minimize transmission through sharps injuries and mucous membranes. Furthermore, after a sharps injury, the health care provider would know to begin post-exposure prophylaxis immediately, without encountering the difficulties of patients’ consent for subsequent HIV testing [3].

In our study, we analyzed the demographic characteristics of HIV-positive patients diagnosed in the First Affiliated Hospital of Harbin Medical University from 2007 to 2012. We found that HIV infection in patients appeared to differ by age, sex, occupation, marital status, educational level, and route of infection.

More than 85% of the HIV-positive patients were 20–60 years old (Table 1). People aged 20–60 years are sexually active; therefore, HIV testing needs to be offered to hospitalized patients in this age group. If a person is found to be HIV positive, they should be counseled to notify their sexual partner and to encourage the partner to be tested for HIV. In September 2006, the Centers for Disease Control and Prevention issued recommendations for HIV testing in health care settings. HIV screening is recommended for patients aged 13–64 years in all health care settings, after informing the patients that testing will be performed, unless the patient declines [14].

It has been reported that 66.5% of newly diagnosed HIV-positive people in Heilongjiang province acquired their infection through sexual transmission [15]. Male-male sexual transmission may be the main mode of HIV transmission in Harbin [16]. Consistent with previous studies, our analysis showed that HIV is common among homosexual men. The majority of men who have sex with men (MSM) in our study were sexually active. Most respondents reported having more than five male sexual partners in the 6 months prior to the survey. These data indicate that the MSM population is highly vulnerable to HIV infection. Homosexual activities face strong negative social pressure in China, which has made the MSM population difficult to reach [17]. HIV screening tests in hospital are convenient and fast, and for this reason we have found that the method is accepted by MSM as a way to learn their HIV status.

This study demonstrated that there is a relationship between marital status and HIV. Individuals who are single constitute a larger proportion of the HIV-infected patients than those who are married. There are several possible explanations for this result. First, it is conceivable that the risk of HIV/AIDS is higher because individuals who are single are more likely to have a higher number of sexual partners throughout their life, leading to greater chances of becoming HIV positive. Second, the MSM population is highly vulnerable to HIV infection. Homosexual men are more likely to choose to live with a male partner and less likely to have sex with women. According to our data, in the single group, the likely route of infection was as follows: homosexual contact (60.5%), heterosexual contact (28.1%), blood (9.7%), unknown (1.8%); in the married group, heterosexual contact was the most common (53.6%) followed by homosexual contact (32.1%), blood (10.7%), and unknown (3.6%) (data not shown).

Over 80% of HIV-positive patients did not have higher education. This study shows that the proportion of HIV-positive patients with higher education increased in the last 3 years of this study, to 17.24%, 16.67% and 30.23%. School-based HIV/AIDS health education can be more efficiently delivered than other programs that prevent the spread of HIV [18]. The prevalence of HIV/AIDS is higher among individuals with lower levels of education [19],[20], and most students with higher education have a good understanding of the means of HIV/AIDS transmission and prevention. However, many people fail to use condoms with steady and casual male partners, putting them at high risk of HIV transmission and infection [21]. The prevalence of HIV among MSM students has reached 3.0%, which is >50 times higher than in the Chinese general population [22]. HIV/AIDS-related knowledge among junior high school students is inadequate, and more attention should be paid to enhance HIV/AIDS-related knowledge, especially among younger students who have not experienced HIV/AIDS-related education [23].

In China, most blue-collar workers have low education and low income. A previous study in China reported that this group had the highest rate for multiple sexual partners, and 90% of blue-collar workers felt they were not at risk of HIV transmission [24]. Other than the employers who have direct and daily contact with workers, organizations including government, schools, and hospitals cannot easily and effectively communicate with adult members of the blue-collar workforce. A high percentage of HIV-positive patients were blue-collar workers, both in our study and a previous study [25]. A survey has shown that 41.7% of migrant workers and 24.4% of blue-collar workers did not know where to go for HIV testing [24]. HIV testing in a hospital is a good way to increase the number of blue-collar workers who know they are infected, and thus have an opportunity for early diagnosis and treatment.

Although HIV prevalence in China is low, Chinese people are facing challenges with respect to HIV/AIDS, including MSM and intravenous drug users with multiple sexual partners, low rate of condom use, needle and syringe sharing, and increased abuse of new drugs. Health promotion and intervention is not easily accomplished among those at high risk, particularly with the annual movement of 220 million people from the countryside to cities in China. As a result, the rural-to-urban migrant population has been difficult to reach with preventive health education, and this population has been deprived of access to health care. Most of the migrants are unmarried or married but living apart from their spouses or children, and with low awareness of HIV/AIDS; a quarter of the newly diagnosed HIV patients are already at the stage of clinical AIDS [26].

## Conclusion

Through HIV testing, uninfected individuals can take steps to avoid becoming infected, while infected individuals can avoid transmission to sexual partners or children. HIV tests are currently available in major hospitals in Chinese cities but are not routinely offered. Offering HIV testing routinely in hospitals is feasible and may increase HIV care and treatment for many individuals.

## Authors’ contributions

H-FX and H-ZZ participated in the study design and drafted the manuscript. L-XJ participated in the data collection and performed the statistical analysis. NZ and XZ carried out the HIV testing. X-RG conceived the study and participated in its design and coordination. All authors read and approved the final manuscript.

## Authors’ information

Co-first author Hai-Zhou Zhou.

## Authors’ original submitted files for images

Below are the links to the authors’ original submitted files for images. Authors’ original file for figure 1 Authors’ original file for figure 2
