# Supplementary figures and images for: Trends in HIV infection in the First Affiliated Hospital of Harbin, China
Source: BMC Infect Dis. 2014 Nov 25;14:605. doi: 10.1186/s12879-014-0605-1 (PMC4245807; doi:10.1186/s12879-014-0605-1)

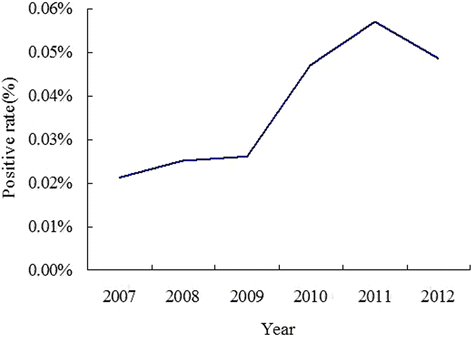

Supplement: Supplementary file 1 — Authors’ original file for figure 1 [file 12879_2014_605_MOESM1_ESM.gif]

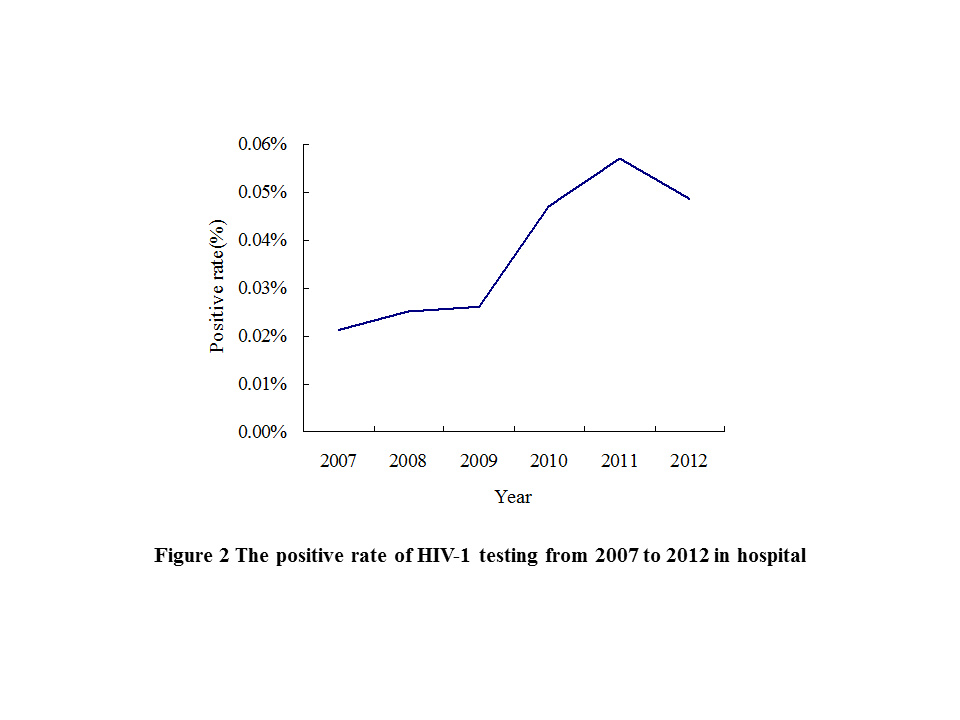

Supplement: Supplementary file 2 — Authors’ original file for figure 2 [file 12879_2014_605_MOESM2_ESM.tiff]
